# The associations of the triglyceride–glucose index and estimated glucose disposal rate with incident cardiometabolic multimorbidity vary across obesity phenotypes: a longitudinal cohort study

**DOI:** 10.3389/fnut.2026.1805727

**Published:** 2026-07-09

**Authors:** Chenyang Li, Yu Feng, Ying Tang, Yupu Shao, Xiaoqin Luo, Yifan Chen, Jiafeng Lin, Shengyuan Gu

**Affiliations:** 1 Department of Cardiology, The Second Affiliated Hospital, Wenzhou Medical University, Wenzhou, China; 2 The Second Xiangya Hospital, Central South University, Changsha, China; 3 Department of Nephrology, The Second Xiangya Hospital of Central South University, Changsha, China; 4 Experimental Center, Department of Basic Medicine, Henan Medical College, Zhengzhou, China; 5 Department of Cardiology, Zhengzhou Central Hospital Affiliated to Zhengzhou University, Zhengzhou, China

**Keywords:** cardiometabolic multimorbidity, eGDR and insulin resistance, insulin resistance, obesity phenotypes, TyG

## Abstract

**Background:**

Cardiometabolic multimorbidity (CMM) is increasingly common in older adults and is closely linked to insulin resistance (IR). However, whether IR surrogates perform similarly across heterogeneous obesity phenotypes remains unclear. Using the English Longitudinal Study of Ageing (ELSA), we examined the phenotype-specific associations of the triglyceride–glucose (TyG) index and the estimated glucose disposal rate (eGDR) with incident CMM and compared their predictive utility.

**Methods:**

We included 4,198 participants from the ELSA in our analysis. Cox proportional hazards models and restricted cubic splines were used to evaluate the association between IR markers (the TyG index and eGDR) and the risk of CMM, as well as to assess potential nonlinearity. We then quantified the IR–related disease burden across obesity phenotypes using the population attributable fraction (PAF) and compared the predictive importance of variables across obesity subgroups using Shapley additive explanations (SHAP) values derived from machine learning models.

**Results:**

Over a median follow-up of 6.8 years, there were 547 incident CMM events. The association between IR and CMM was strongest in the predominantly isolated central obesity group, surpassing those observed in the no obesity-risk and dual obesity-risk groups. In this subgroup, each 1-standard deviation (SD) increase in the TyG index was linked to a 60.8% higher risk of incident CMM, whereas each 1-SD increase in eGDR was linked to a 60.1% lower risk. Restricted cubic spline analyses revealed monotonic, linear associations for both TyG and eGDR across all three obesity phenotype groups. PAF analyses further showed that the predominantly isolated central obesity group had the highest attributable burden for both TyG and eGDR (37 and 65%, respectively). In internal validation, the machine learning models demonstrated promising discriminative ability, with areas under the curve of 0.827, 0.945, and 0.881. SHAP analyses identified eGDR and TyG as the leading contributors to model predictions.

**Conclusion:**

IR surrogates were associated with incident CMM and showed potential utility for CMM risk prediction, with the strongest gradients and greatest population impact observed in the predominantly isolated central obesity phenotype. eGDR may be particularly useful for phenotype-tailored CMM risk stratification.

## Graphical abstract

**Figure:**
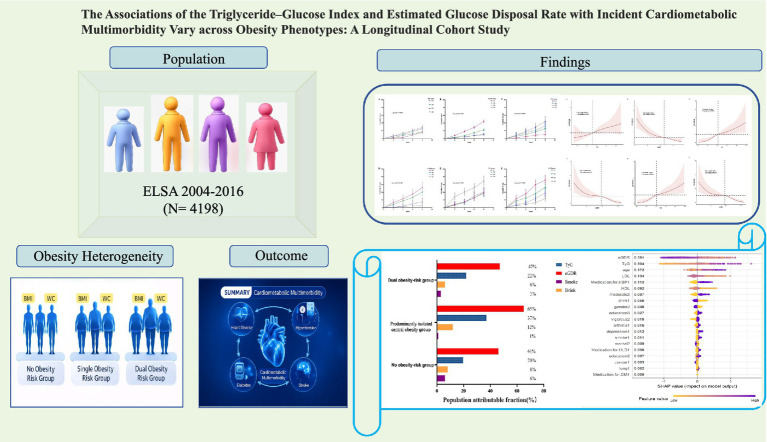

## Introduction

Cardiometabolic multimorbidity (CMM), characterized by the clustering of at least two major conditions such as type 2 diabetes, coronary heart disease, or stroke, imposes a growing strain on global healthcare infrastructure (1–3). Its prevalence is on the rise, partly attributable to population aging. According to data from 1999 to 2018 in the US, the prevalence of CMM has been steadily increasing, reaching 14.4% in 2017–2018 (4). Crucially, CMM confers a far graver prognosis than individual cardiometabolic diseases, being linked to a multiplicative mortality risk and an up to 12-year estimated loss in life expectancy (5–7).

Beyond the well-established role of overweight and obesity as hazardous factors for individual cardiometabolic conditions (8, 9), insulin resistance (IR) is increasingly identified as the core mechanism responsible for both the development of individual conditions (10, 11) and their convergence into multimorbidity (12). However, the application of IR assessment faces feasibility constraints. The hyperinsulinemic–euglycemic clamp, while considered the gold standard for direct IR measurement, is often impractical due to its complexity and resource intensity (13). This limitation has accelerated the development of accessible surrogate markers. Among these, the triglyceride-glucose (TyG) index, based on fasting levels of triglycerides and glucose, is a robust and convenient indicator of hepatic and systemic IR, with the major advantage of utilizing routine laboratory parameters for high clinical utility and reproducibility (14, 15). In parallel, the estimated glucose disposal rate (eGDR), calculated from waist circumference, hypertension status, and glycated hemoglobin (HbA1c), provides a distinct yet complementary measure that reflects peripheral insulin sensitivity and integrated long-term metabolic and vascular health (16–19). Notably, both TyG and eGDR are independent predictors of CMM risk (20–22).

The evolving conceptualization of obesity introduces a critical yet largely unexplored dimension in understanding its relationship with CMM. A paradigm shift is underway, redefining obesity beyond body mass index (BMI) to include anthropometric measures (e.g., waist circumference (WC) and waist-to-height ratio) and direct adiposity assessments. This new framework further stratifies obesity into preclinical and clinical stages based on complication status (23–25), which aims to more accurately identify individuals with pathological adiposity and their associated higher health risks (24). Given the established role of IR as a key mediator driving obesity to cardiometabolic diseases (26), a pivotal question arises: Does the predictive value of IR for CMM vary across these newly defined obesity subgroups? Elucidating this relationship is essential to determine whether IR assessment can refine risk stratification within the new obesity subgroups and to identify which subgroup populations may benefit most from targeted therapies designed to improve insulin sensitivity to prevent CMM.

To address this gap, we evaluated the relationship between two surrogate IR markers (the TyG index and eGDR) and the incidence of CMM across newly defined obesity subgroups (no obesity-risk group, predominantly isolated central obesity group, and dual obesity-risk group). We combined conventional Cox proportional hazards regression with machine learning methods to evaluate the linkage between IR and co-occurring cardiometabolic conditions within these obesity phenotypes. Given that machine learning is adept at handling large datasets and highly correlated predictors, we used Shapley additive explanations (SHAP) values to measure the contribution of each variable to the model’s predictions (27). Our analysis aims to clarify the role of IR within the newly defined obesity subgroups and to evaluate the potential of TyG and eGDR as practical tools for refining CMM risk assessment in specific high-risk populations.

## Methods

### Study design and participants

We utilized data from the English Longitudinal Study of Ageing (ELSA), a longitudinal survey in England. The study consistently collects information on demographic characteristics, socioeconomic circumstances, and health-related factors (28). We analyzed data spanning Waves 2 to 8 (2004–2016). From an initial cohort of 17,070 participants aged ≥50 years, we excluded 11,374 individuals with insufficient data to calculate the TyG index, eGDR, BMI, or WC. We then removed 1,498 participants who either already met the definition of CMM at baseline (Wave 4) or lacked baseline and/or follow-up data on these conditions. The final analytic sample included 4,198 participants; the selection process is summarized in Supplementary Figure 1.

### Obesity phenotypes

Following World Health Organization guidelines, which consider BMI and WC thresholds and their variation across populations, we defined an adiposity-risk phenotype using both BMI and WC. A high BMI was defined as BMI ≥ 30 kg/m^2^, whereas a high WC was defined as >102 cm in men and >88 cm in women (23, 24, 29). Participants were categorized into three groups: (1) no obesity-risk group, where neither BMI nor WC met the obesity threshold; (2) single obesity-risk group, where either BMI or WC met the threshold; and (3) dual obesity-risk group, where both BMI and WC met the threshold (Supplementary Table 1). Because this single obesity-risk group consisted predominantly of participants with elevated WC and BMI < 30 kg/m^2^ (97%), it is hereafter referred to as the predominantly isolated central obesity group.

### IR–related measures

We focused on two widely used markers of metabolic impairment: the TyG index and the eGDR; formulas are provided in Supplementary Materials (30, 31). Because blood-based measures are not available for all participants in any single wave of ELSA, relying on one wave would markedly reduce the usable sample size. To maximize the number of participants with both measures, we combined data from Waves 2 and 4 and analyzed the earliest available measurement for each participant.

### Definition of CMM

CMM was defined as the presence of at least two of the following conditions: heart disease, hypertension, stroke, or diabetes. Heart disease, hypertension, and stroke were identified from reported physician diagnoses, while diabetes was determined based on physician diagnosis and/or the use of glucose-lowering medication. For each condition, the first recorded diagnosis date was regarded as the onset. Details are offered in Supplementary Materials. Although death can preclude the observation of incident multimorbidity, only 45 deaths were noted within this cohort. Given the limited number of events, modelling approaches that treat death as an alternative event (e.g., the Fine–Gray subdistribution hazards framework) or analyzing death as a secondary endpoint would yield unstable estimates; therefore, death was not included as a secondary outcome.

### Covariates

Covariates were defined at baseline (Wave 4) and covered sociodemographic factors (age, sex, marital status, and educational attainment), measures of adiposity and lipids [BMI, WC, low-density lipoprotein cholesterol (LDL-C), and high-density lipoprotein cholesterol (HDL-C)], and health-related behaviours (smoking status, alcohol use, and the frequency of moderate and vigorous physical activity). We also accounted for selected comorbid conditions (chronic lung disease, cancer, rheumatoid arthritis, and depression) and medication use related to hypertension, dyslipidaemia, and diabetes. Comorbidities were identified through reports of physician-diagnosed conditions, and all covariates were anchored to the Wave 4 assessment as baseline. Missingness was under 10% for most covariates; however, LDL-C had a substantially higher proportion of missing data (38%; Supplementary Table 3). Missing values were imputed using a random forest–based approach, under the assumption that the data were missing at random conditional on observed covariates.

### Statistical analysis

Following established practice, we compared groups using statistical tests appropriate to the type and distribution of each baseline variable; details are provided in Supplementary Methods (30). Supplementary Figure 2 shows the distributions of TyG and eGDR across obesity subgroups.

To examine the associations of IR–related measures (TyG and eGDR) with incident CMM, we fitted Cox regression models. Prior to model fitting, multicollinearity among candidate covariates was checked using variance inflation factors (VIFs); variables with a VIF of ≥5 were excluded from the main model to improve numerical stability.

Based on the VIF screening, the main multivariable model included 17 covariates, encompassing sociodemographic factors, health behaviours, lipid measures, cardiometabolic medication use, and history of comorbidities (Supplementary Table 5). BMI and WC were excluded due to their VIF exceeding 5.

To assess robustness, we fitted three progressively adjusted Cox models: Model 1 included demographic factors; Model 2 additionally accounted for lifestyle factors; and Model 3 further incorporated comorbidities, lipid measures, and medication use. The proportional hazards assumption was assessed using Schoenfeld residuals, and all models met this assumption. Kaplan–Meier (K-M) curves were plotted and compared using the log-rank test. We conducted subgroup analyses by sex and by age (<65 vs. ≥ 65 years) to explore potential differences in effect across groups. To evaluate possible non-linear exposure–outcome relationships, we modelled TyG and eGDR using restricted cubic splines (RCS) with four knots. We further estimated population attributable fractions (PAFs) for CMM based on adjusted hazard ratios, following approaches described previously (32). Because TyG and eGDR could come from different survey waves for some participants, we performed a sensitivity analysis restricted to those with both TyG and eGDR measured in Wave 4.

In addition, we built a prediction model using eXtreme Gradient Boosting (XGBoost) with the VIF-screened variables. Model training and hyperparameter tuning were conducted using five repeats of five-fold cross-validation. Discrimination was primarily assessed using the cross-validated mean area under the receiver operating characteristic curve (AUROC), with sensitivity, specificity, positive predictive value (PPV), and negative predictive value (NPV) reported as complementary measures. To improve interpretability, we computed SHAP values and visualised feature contributions, quantifying each variable’s average marginal contribution based on cooperative game theory.

*p* < 0.05 was considered statistically significant. All analyses were conducted in R (version 4.3.2).

## Results

### Baseline characteristics

Among the 4,198 participants: 2,180 were in the no obesity-risk group, 966 were in the predominantly isolated central obesity group, and 1,052 were in the dual obesity-risk group. Baseline characteristics are summarized in Table 1. Overall, participants in the predominantly isolated central obesity and dual obesity-risk groups were more frequently women, had lower educational attainment, and reported less physical activity than those in the no obesity-risk group (all *p* < 0.001). Among the assessed comorbidities, rheumatoid arthritis was more common in the dual obesity-risk categories. Metabolic profiles also differed across groups: the predominantly isolated central obesity and dual obesity-risk groups showed larger WC and BMI, higher triglycerides (TG), fasting plasma glucose (FPG), HbA1c, and TyG index, along with lower HDL-C and eGDR (Table 1). Baseline differences between excluded participants and the analytic sample are reported in Supplementary Table 2.

**Table 1:** Baseline clinical characteristics according to the presence of obesity-risk phenotypes.

Variables	Total (*n* = 4,198)	No obesity-risk group (*n* = 2,180)	Predominantly isolated central obesity group (*n* = 966)	Dual obesity-risk group (*n* = 1,052)	*p* value
Age, median (IQR)	63.0 (58.0, 70.0)	63.0 (58.0, 70.0)	64.0 (59.0, 71.0)	63.0 (58.0, 70.0)	**0.003**
Gender, *n* (%)					**<0.001**
Male	1,860 (44.3)	1,078 (49.4)	373 (38.6)	409 (38.9)	
Female	2,338 (55.7)	1,102 (50.6)	593 (61.4)	643 (61.1)	
Education, *n* (%)					**<0.001**
Less than high school	1,402 (33.4)	608 (27.9)	357 (37)	437 (41.5)	
High school or equivalent	1,006 (24.0)	529 (24.3)	245 (25.4)	232 (22.1)	
College or above	1,790 (42.6)	1,043 (47.8)	364 (37.7)	383 (36.4)	
Marital, *n* (%)					0.373
Married or partnered	3,136 (74.7)	1,643 (75.4)	724 (74.9)	769 (73.1)	
Divorced or never partnered	1,062 (25.3)	537 (24.6)	242 (25.1)	283 (26.9)	
Smoke, *n* (%)					**0.005**
No	3,683 (87.7)	1,884 (86.4)	848 (87.8)	951 (90.4)	
Yes	515 (12.3)	296 (13.6)	118 (12.2)	101 (9.6)	
Drink, *n* (%)					**<0.001**
No	1,228 (29.3)	541 (24.8)	290 (30)	397 (37.7)	
≥1 days/week	2,970 (70.7)	1,639 (75.2)	676 (70)	655 (62.3)	
Moderate physical activity, *n* (%)					**<0.001**
≥1 days/week	2,930 (69.8)	1,643 (75.4)	644 (66.7)	643 (61.1)	
<1 days/week	1,268 (30.2)	537 (24.6)	322 (33.3)	409 (38.9)	
Vigorous physical activity, *n* (%)					**<0.001**
≥1 days/week	1,025 (24.4)	630 (28.9)	198 (20.5)	197 (18.7)	
<1 days/week	3,173 (75.6)	1,550 (71.1)	768 (79.5)	855 (81.3)	
BMI, Median (IQR)	26.9 (24.5, 30.2)	24.8 (23.0, 26.4)	27.8 (26.6, 29.1)	32.7 (31.1, 35.3)	**<0.001**
WC, Median (IQR)	94.0 (85.5, 102.7)	85.9 (79.7, 93.9)	97.0 (91.3, 104.1)	107.6 (100.5, 113.5)	**<0.001**
TyG, Median (IQR)	7.0 (6.6, 7.3)	6.8 (6.5, 7.1)	7.0 (6.8, 7.4)	7.2 (6.9, 7.5)	**<0.001**
eGDR, Median (IQR)	8.4 (6.1, 10.0)	9.6 (7.3, 10.7)	7.9 (5.8, 9.5)	5.8 (4.7, 8.2)	**<0.001**
TG, Median (IQR)	1.4 (1.0, 1.9)	1.2 (0.9, 1.7)	1.5 (1.1, 2.0)	1.6 (1.2, 2.3)	**<0.001**
FPG, Median (IQR)	4.8 (4.5, 5.1)	4.7 (4.4, 5.0)	4.8 (4.5, 5.1)	4.9 (4.5, 5.3)	**<0.001**
Hba1c, Median (IQR)	5.7 (5.5, 5.9)	5.7 (5.5, 5.9)	5.7 (5.5, 6.0)	5.8 (5.6, 6.1)	**<0.001**
HDL, Median (IQR)	1.5 (1.3, 1.8)	1.6 (1.4, 1.9)	1.5 (1.3, 1.8)	1.4 (1.2, 1.7)	**<0.001**
LDL, Median (IQR)	3.8 (3.2, 4.4)	3.7 (3.1, 4.3)	3.9 (3.2, 4.5)	3.8 (3.2, 4.4)	0.052
Depression, *n* (%)					**<0.001**
No	3,760 (89.6)	1,995 (91.5)	866 (89.6)	899 (85.5)	
Yes	438 (10.4)	185 (8.5)	100 (10.4)	153 (14.5)	
Cancer, *n* (%)					0.377
No	3,897 (92.8)	2,029 (93.1)	887 (91.8)	981 (93.3)	
Yes	301 (7.2)	151 (6.9)	79 (8.2)	71 (6.7)	
Lung, *n* (%)					0.392
No	4,016 (95.7)	2,094 (96.1)	918 (95)	1,004 (95.4)	
Yes	182 (4.3)	86 (3.9)	48 (5)	48 (4.6)	
Arthritis, *n* (%)					**<0.001**
No	2,839 (67.6)	1,588 (72.8)	635 (65.7)	616 (58.6)	
Yes	1,359 (32.4)	592 (27.2)	331 (34.3)	436 (41.4)	
Medication for HBP, *n* (%)					**<0.001**
No	3,253 (77.5)	1,801 (82.6)	722 (74.7)	730 (69.4)	
Yes	945 (22.5)	379 (17.4)	244 (25.3)	322 (30.6)	
Medication for DM, *n* (%)					**<0.001**
No	4,150 (98.9)	2,166 (99.4)	955 (98.9)	1,029 (97.8)	
Yes	48 (1.1)	14 (0.6)	11 (1.1)	23 (2.2)	
Medication for HLD, *n* (%)					**<0.001**
No	3,481 (82.9)	1,854 (85)	794 (82.2)	833 (79.2)	
Yes	717 (17.1)	326 (15)	172 (17.8)	219 (20.8)	

A *p* value < 0.05 indicated a significant difference. BMI, body mass index; DM, diabetes mellitus; eGDR, estimated glucose disposal rate; FPG, fasting blood glucose; Hba1c, glycosylated hemoglobin; HBP, hypertension; HDL, high-density lipoprotein; HLD, hyperlipidemia; LDL, low-density lipoprotein; IQR, interquartile range; TG, triglyceride; TyG, triglyceride-glucose; WC, waist circumference. All bold text has a *P*-value less than or equal to 0.05, therefore bolding indicates significance.

### Associations between metabolic indices and incident CMM

**Figure 1:**
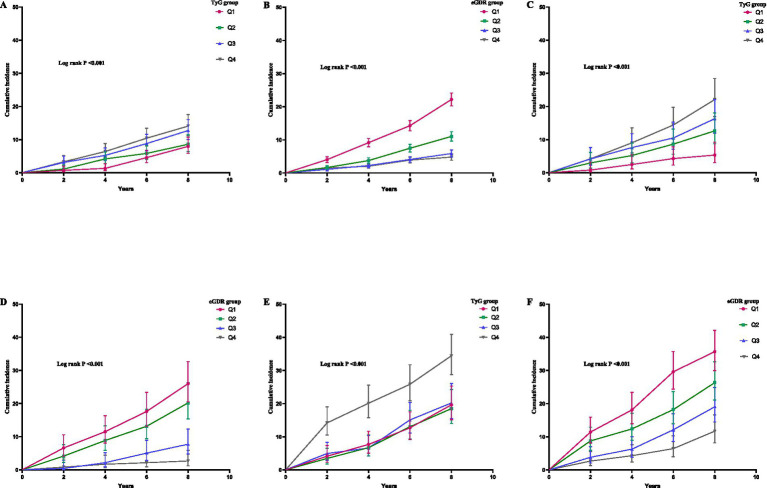
Kaplan–Meier curves for CMM by insulin-resistance indicators (TyG and eGDR). **(A, B)** No obesity-risk group. **(C, D)** Predominantly isolated central obesity group. **(E, F)** Dual obesity-risk group. CMM, cardiometabolic multimorbidity; eGDR, estimated glucose disposal rate; TyG, triglyceride-glucose.

The median follow-up duration was 6.8 years, during which 547 incident CMM events were recorded. The most frequent CMM combination was heart disease plus hypertension (302 events; 55.2%), followed by diabetes plus hypertension (117 events; 21.4%; Supplementary Figure 3). K-M curves showed TyG and eGDR differed across adiposity-phenotype groups (log-rank *p* < 0.001). The highest TyG quartile (Q4) showed the greatest cumulative incidence of CMM, whereas the lowest eGDR quartile (Q1) had the highest risk (Figure 1).

In Model 3, associations were consistent across phenotypes. In the no obesity-risk group, each 1-SD increase in TyG was linked to a higher CMM risk [hazard ratio (HR) 1.201, 95% confidence interval (CI) 1.024–1.409], while each 1-SD increase in eGDR was linked to a lower risk (HR 0.516, 95% CI 0.417–0.638). In the predominantly isolated central obesity group, the corresponding estimates were HR 1.608 (95% CI 1.325–1.952) for TyG and HR 0.399 (95% CI 0.289–0.550) for eGDR. In the dual obesity-risk group, TyG remained positively linked to CMM (HR 1.289, 95% CI 1.113–1.493), whereas eGDR remained inversely linked (HR 0.653, 95% CI 0.535–0.796). These patterns persisted across stepwise adjustment sets (Table 2). A significant interaction was observed between the insulin resistance marker and obesity phenotype for incident CMM (*p* for interaction = 0.04), indicating heterogeneity in the association across the three groups. The association appeared more pronounced in the predominantly isolated central obesity group. Results were similar in sensitivity analyses restricted to participants with both TyG and eGDR measured at Wave 4 (Supplementary Table 4). Using ROC curve analysis, we compared the predictive performance of the TyG index and eGDR for CMM across obesity phenotype subgroups. Across the no obesity-risk, predominantly isolated central obesity group, and dual obesity-risk groups, eGDR consistently showed better discrimination than the TyG index. Notably, both markers performed best in the predominantly isolated central obesity group (Supplementary Figure 4).

**Table 2:** Multivariable associations between metabolic indicators (TyG and eGDR Index) and risk of CMM.

Indices	Groups	Number	Model 1	Model 2	Model 3
			HR (95% CI) *p* value	HR (95% CI) *p* value	HR (95% CI) *p* value
No obesity-risk group					
TyG	Continuous	*N* = 2,180	**1.262 (1.094–1.456) 0.001**	**1.191 (1.027–1.382) 0.021**	**1.201 (1.024–1.409) 0.024**
	Q1	*N* = 545	1(Ref)	1(Ref)	1(Ref)
	Q2	*N* = 545	1.05 (0.680–1.640) 0.814	0.98 (0.633–1.530) 0.942	1.01 (0.641–1.600) 0.956
	Q3	*N* = 545	1.49 (0.996–2.240) 0.052	1.31 (0.867–1.980) 0.199	1.40 (0.899–2.160) 0.137
	Q4	*N* = 545	**1.72 (1.150–2.570) 0.007**	1.48 (0.979–2.230) 0.062	**1.58 (1.000–2.490) 0.049**
	*p* for trend		**<0.001**	**0.024**	**0.018**
eGDR	Continuous	*N* = 2,180	**0.465 (0.388–0.559) < 0.001**	**0.472 (0.389–0.572) < 0.001**	**0.516 (0.417–0.638) < 0.001**
	Q1	*N* = 545	1(Ref)	1(Ref)	1(Ref)
	Q2	*N* = 545	**0.51 (0.366–0.714) < 0.001**	**0.52 (0.373–0.731) < 0.001**	**0.61 (0.431–0.876) 0.007**
	Q3	*N* = 545	**0.30 (0.199–0.463) < 0.001**	**0.32 (0.207–0.487) < 0.001**	**0.41 (0.259–0.655) < 0.001**
	Q4	*N* = 545	**0.26 (0.160–0.426) < 0.001**	**0.28 (0.166–0.478) < 0.001**	**0.34 (0.200–0.605) < 0.001**
	*p* for trend		**<0.001**	**<0.001**	**<0.001**
Predominantly isolated central obesity group					
TyG	Continuous	*N* = 966	**1.563 (1.321–1.848) < 0.001**	**1.582 (1.329–1.884) < 0.001**	**1.608 (1.325–1.952) < 0.001**
	Q1	*N* = 248	1(Ref)	1(Ref)	1(Ref)
	Q2	*N* = 237	**2.28 (1.150–4.530) 0.018**	**2.11 (1.060–4.200) 0.034**	**2.82 (1.350–5.920) 0.005**
	Q3	*N* = 241	**3.13 (1.620–6.030) < 0.001**	**2.88 (1.490–5.570) 0.001**	**3.96 (1.910–8.220) < 0.001**
	Q4	*N* = 240	**4.09 (2.160–7.740) < 0.001**	**3.90 (2.050–7.410) < 0.001**	**4.66 (2.250–9.650) < 0.001**
	*p* for trend		**<0.001**	**<0.001**	**<0.001**
eGDR	Continuous	*N* = 966	**0.389 (0.293–0.515) < 0.001**	**0.388 (0.292–0.516) < 0.001**	**0.399 (0.289–0.550) < 0.001**
	Q1	*N* = 242	1(Ref)	1(Ref)	1(Ref)
	Q2	*N* = 241	0.76 (0.430–1.350) 0.355	0.72 (0.414–1.250) 0.243	0.57 (0.330–1.010) 0.056
	Q3	*N* = 241	**0.28 (0.156–0.488) < 0.001**	**0.26 (0.146–0.458) < 0.001**	**0.29 (0.163–0.540) < 0.001**
	Q4	*N* = 242	**0.11 (0.042–0.281) < 0.001**	**0.11 (0.043–0.285) < 0.001**	**0.08 (0.031–0.249) < 0.001**
	*p* for trend		**<0.001**	**<0.001**	**<0.001**
Dual obesity-risk group					
TyG	Continuous	*N* = 1,052	**1.359 (1.194–1.547) < 0.001**	**1.334 (1.168–1.523) < 0.001**	**1.289 (1.113–1.493) < 0.001**
	Q1	*N* = 267	1(Ref)	1(Ref)	1(Ref)
	Q2	*N* = 261	0.92 (0.603–1.390) 0.679	0.89 (0.584–1.35) 0.576	0.88 (0.575–1.370) 0.586
	Q3	*N* = 263	1.05 (0.700–1.580) 0.805	1.02 (0.676–1.530) 0.935	1.03 (0.668–1.600) 0.880
	Q4	*N* = 261	**2.06 (1.430–2.960) < 0.001**	**1.96 (1.360–2.830) < 0.001**	**1.74 (1.160–2.610) 0.007**
	*p* for trend		**<0.001**	**<0.001**	**0.002**
eGDR	Continuous	*N* = 1,052	**0.553 (0.465–0.657) < 0.001**	**0.535 (0.437–0.655) < 0.001**	**0.653 (0.535–0.796) < 0.001**
	Q1	*N* = 263	1(Ref)	1(Ref)	1(Ref)
	Q2	*N* = 263	**0.65 (0.469–0.913) 0.012**	**0.65 (0.449–0.937) 0.021**	**0.70 (0.499–0.985) 0.040**
	Q3	*N* = 263	**0.44 (0.307–0.644) < 0.001**	**0.44 (0.297–0.654) < 0.001**	**0.57 (0.387–0.846) 0.005**
	Q4	*N* = 263	**0.28 (0.178–0.435) < 0.001**	**0.28 (0.168–0.452) < 0.001**	**0.43 (0.265–0.710) < 0.001**
	*p* for trend		**<0.001**	**<0.001**	**<0.001**

A *p* value < 0.05 indicated a significant difference. BMI, body mass index; CMM, cardiometabolic multimorbidity; DM, diabetes mellitus; eGDR, estimated glucose disposal rate; FPG, fasting blood glucose; Hba1c, glycosylated hemoglobin; HBP, hypertension; HDL, high-density lipoprotein; HLD, hyperlipidemia; LDL, low-density lipoprotein; TG, triglyceride; TyG, triglyceride-glucose. Model 1: adjusted for age, sex, marital status, and education. Model 2: adjusted for age, sex, marital status, education, smoking status, alcohol consumption, vigorous physical activity, and moderate physical activity. Model 3: adjusted for age, sex, marital status, education, smoking status, alcohol consumption, vigorous physical activity, moderate physical activity, LDL, HDL, medication for HBP, medication for DM, medication for HLD, lung disease, cancer, arthritis and depression. All bold text has a *P*-value less than or equal to 0.05, therefore bolding indicates significance.

**Figure 2:**
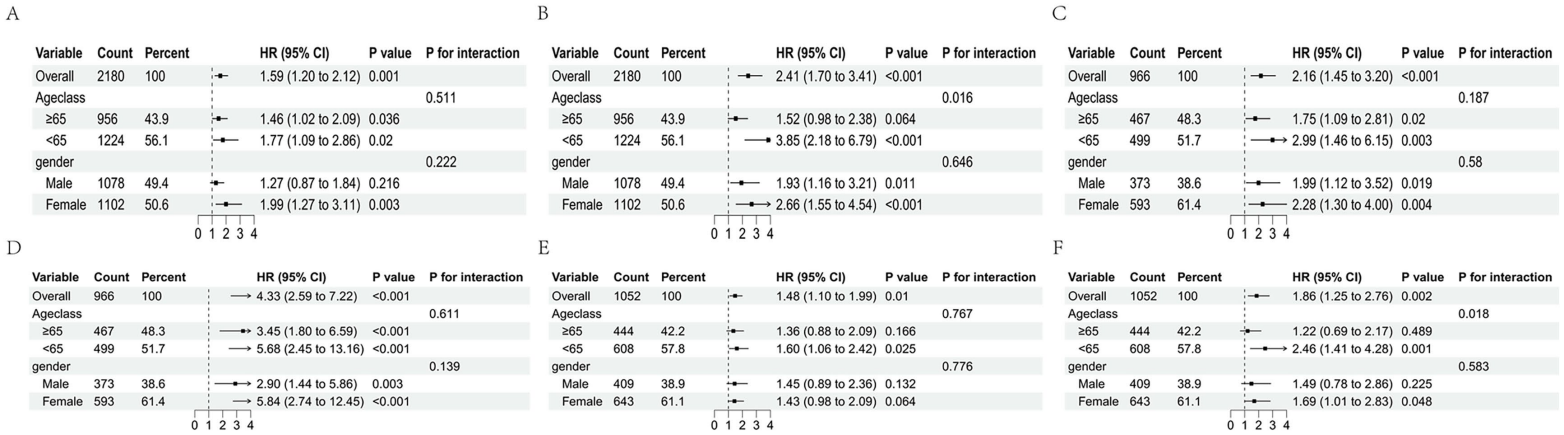
Subgroup analyses of associations between insulin-resistance indicators (TyG and eGDR) and CMM risk. **(A, B)** No obesity-risk group. **(C, D)** Predominantly isolated central obesity group. **(E, F)** Dual obesity-risk group. CMM, cardiometabolic multimorbidity; eGDR, estimated glucose disposal rate; TyG, triglyceride-glucose.

Across subgroups, the protective gradient for eGDR appeared more pronounced for CMM among participants aged <65 years in both the no obesity-risk and dual obesity-risk groups. The findings were comparable between men and women, with no statistically significant differences (Figure 2).

### Shape of the associations with incident CMM

**Figure 3:**
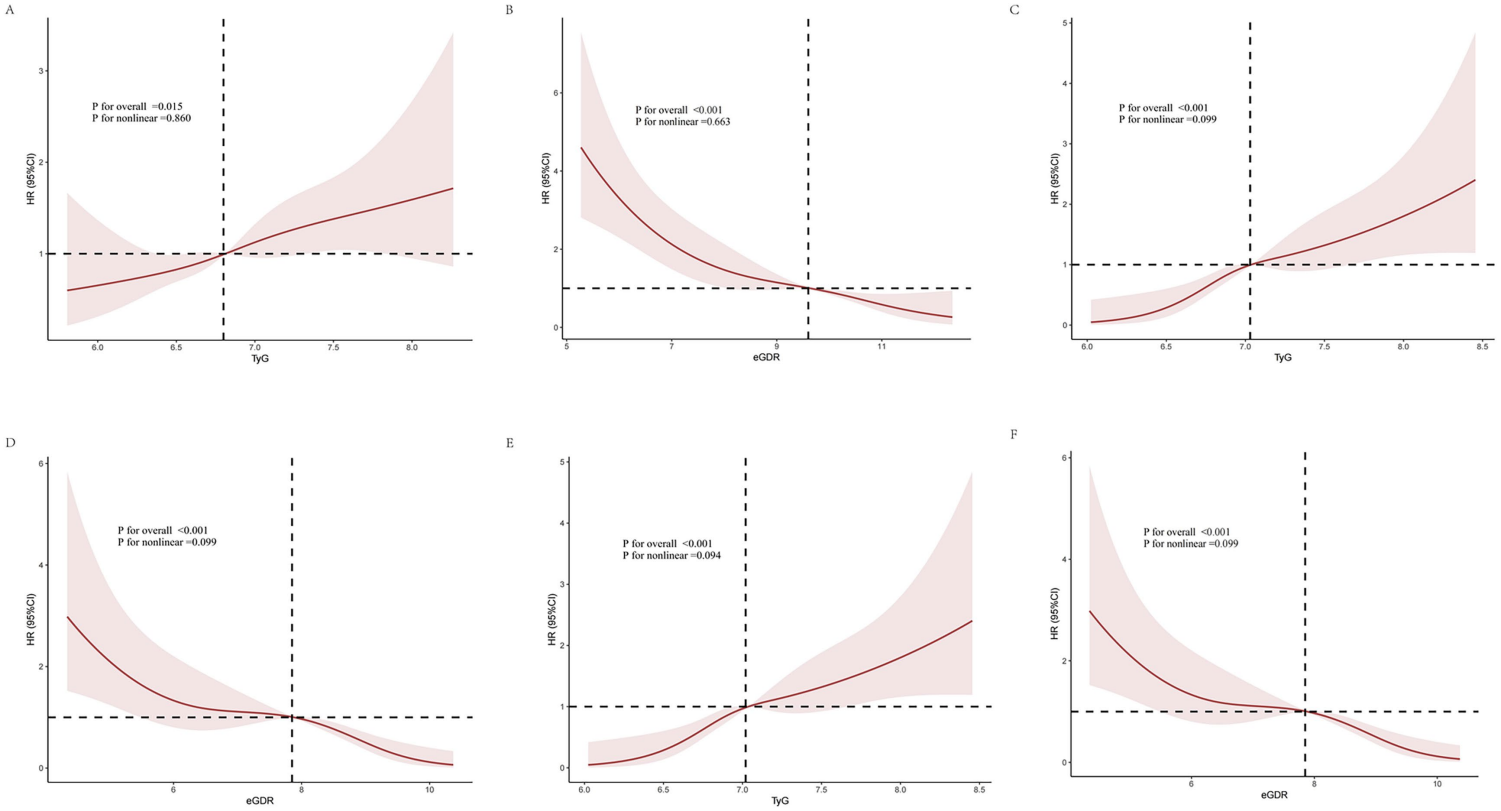
Nonlinear associations of insulin-resistance indicators (TyG and eGDR) with CMM. **(A, B)** No obesity-risk group. **(C, D)** Predominantly isolated central obesity group. **(E, F)** Dual obesity-risk group. CMM, cardiometabolic multimorbidity; eGDR, estimated glucose disposal rate; TyG, triglyceride-glucose.

RCS analyses indicated a predominantly linear, one-directional pattern across all adiposity-phenotype groups: higher TyG was linked to higher CMM risk, whereas higher eGDR was linked to lower risk. No clear departure from linearity was observed (Figure 3).

### Population impact estimates

**Figure 4:**
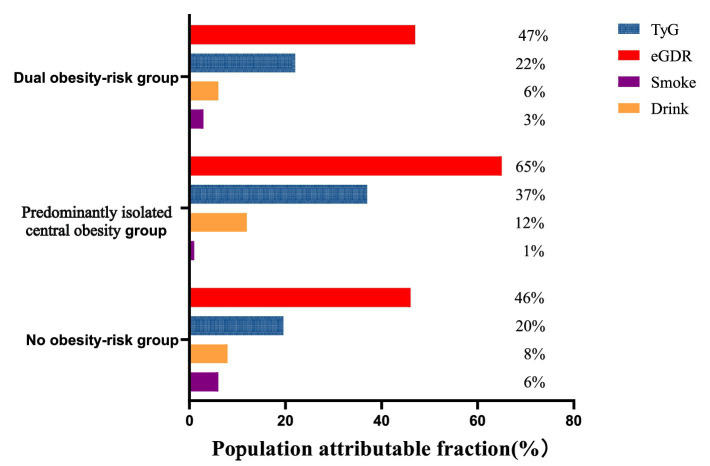
Population attribution fraction for metabolic indicators. eGDR, estimated glucose disposal rate; TyG, triglyceride-glucose.

PAF estimates suggested that the population-level contribution of IR–related measures to incident CMM differed across adiposity phenotypes. In the no obesity-risk group, the PAFs for TyG and eGDR were 20 and 46%, respectively. In the predominantly isolated central obesity group, the corresponding PAFs were 37 and 65%. In the dual obesity-risk group, the PAFs were 22 and 47%. Overall, across phenotypes, the preventable proportion of CMM linked to TyG and eGDR appeared greater than that linked to traditional behavioural risk factors (smoking and alcohol use). Notably, the predominantly isolated central obesity group showed the highest PAFs for both TyG and eGDR, exceeding those observed in the no obesity-risk and dual obesity-risk groups (Figure 4).

### Machine-learning–based assessment of feature importance

Pairwise correlations among candidate predictors are shown in Supplementary Figure 5. HDL-C was inversely linked to the TyG index, while BMI and WC were inversely linked to eGDR. In contrast, LDL-C, BMI, and WC showed positive correlations with the TyG index.

Prediction models developed for each adiposity-phenotype subgroup showed strong discrimination. Using the chosen operating thresholds, the performance metrics in the no obesity-risk group were as follows: cutoff 0.361, AUROC 0.827, sensitivity 0.714, specificity 0.815, PPV 0.292, and NPV 0.964. In the predominantly isolated central obesity group, the corresponding values were: cutoff 0.246, AUROC 0.945, sensitivity 0.840, specificity 0.945, PPV 0.684, and NPV 0.976. In the dual obesity-risk group, the performance metrics were: cutoff 0.342, AUROC 0.881, sensitivity 0.802, specificity 0.834, PPV 0.559, and NPV 0.941 (Table 3).

**Table 3:** Performance of the three machine-learning models.

Model	Cutoff*	AUC	Sensitivity	Specificity	PPV	NPV
No obesity-risk group	0.361211985	0.827	0.7142857	0.815736	0.2923977	0.9640072
Predominantly isolated central obesity group	0.246352062	0.945	0.8403361	0.9456907	0.6849315	0.9768293
Dual obesity-risk group	0.342098802	0.881	0.8027523	0.8345324	0.5591054	0.9418133

*Cutoff values were determined using the Youden index, defined as sensitivity + specificity − 1. AUC, area under the curve; NPV, negative predictive value; PPV, positive predictive value.

**Figure 5:**
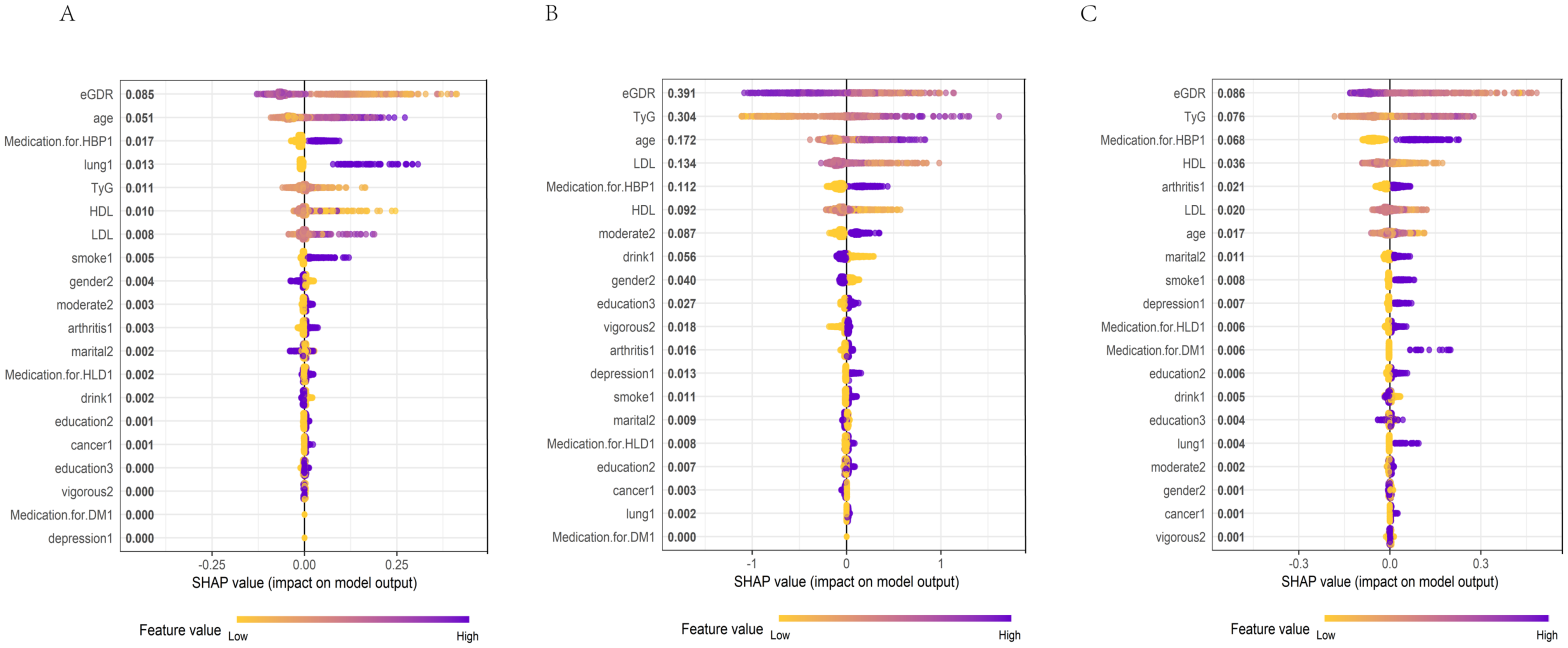
SHAP summary plot for the prediction model. **(A)** No obesity-risk group. **(B)** Predominantly isolated central obesity group. **(C)** Dual obesity-risk group. eGDR, estimated glucose disposal rate; SHAP, Shapley additive explanations; TyG, triglyceride-glucose.

Figure 5 presents SHAP summary plots. In all three subgroup models, eGDR and the TyG index consistently ranked as the top predictors, with especially pronounced contributions observed in the predominantly isolated central obesity group.

## Discussion

The present study demonstrated a significant linkage between surrogate markers of IR (TyG and eGDR) and the incidence of CMM, identifying them as reliable predictive factors. Notably, this linkage exhibited marked heterogeneity across redefined obesity subcategories. Specifically, our results showed that among individuals in the predominantly isolated central obesity group, the independent effects of TyG and eGDR on CMM incidence were most pronounced, suggesting that modulating IR in this subpopulation could yield substantial benefits in reducing CMM risk, a conclusion further supported by our PAF analysis. Collectively, these findings provide novel evidence that within a newly refined obesity classification, TyG and eGDR remain effective predictors of CMM, with their predictive strength peaking in the predominantly isolated central obesity group. This may help identify subgroups in whom future studies of interventions targeting insulin sensitivity and CMM prevention could be prioritized.

Our findings align with and substantially extend the increasing findings underscoring the independent predictive value of the TyG index and the eGDR for CMM (7, 33–35). In a large-scale analysis involving 304,568 UK Biobank participants, Liu et al. demonstrated that a higher Life’s Essential 8 (LE8) score, where TyG and CRP contributed significantly to its protective effect, was linked to a markedly lower risk of CMM, emphasizing the pivotal role of IR in its pathogenesis (12). Similarly, Ruan et al. reported that the combination of high TyG-related indices and a low eGDR nearly tripled CMM incidence, further identifying eGDR as a central mediator of the effects conveyed by TyG-based markers (36). Adding another dimension, Shi et al. provided mechanistic evidence that long-term exposure to ambient particulate matter elevates CMM risk, a relationship partly mediated through increased TyG levels (37).

Crucially, our study advances this established knowledge by revealing a critical nuance: the predictive strength of TyG and eGDR is not uniform across newly refined obesity populations. Notably, the predictive strength of the markers was markedly stronger in the predominantly isolated central obesity group. This apparent paradox can be understood by recognizing the distinct roles of general adiposity (captured by BMI) and central adiposity (reflected by WC) in cardiometabolic risk. While BMI represents overall body mass, it fails to differentiate between fat and muscle or to account for fat distribution. In contrast, WC specifically measures abdominal, particularly visceral fat, which is metabolically active and strongly linked to IR, inflammation, and dyslipidemia (38). Evidence from a recent analysis by Cho et al. suggests that BMI and central obesity play differential roles in modulating fracture risk, offering a relevant analogy for understanding cardiometabolic outcomes (39). In their analysis, a higher BMI was generally protective against fractures, yet central obesity, indicated by elevated WC or waist-to-height ratio, was independently concordant with increased fracture risk, especially among individuals with overweight or obesity. This dissociation highlights that BMI alone may mask the detrimental metabolic effects of visceral fat accumulation.

In our study, the predominantly isolated central obesity group, comprising approximately 97% of participants with normal BMI but elevated WC, and 3% with elevated BMI but normal WC, demonstrates that central obesity acts as the dominant metabolic stressor. The comparatively weaker association observed in the dual obesity-risk group should be interpreted cautiously. Participants with both elevated BMI and waist circumference may already have a high baseline cardiometabolic risk, thereby reducing the relative discriminatory contribution of insulin resistance markers. Restricted within-group variability, differences in sample size or event numbers, and residual confounding may also have contributed to the observed pattern.

To compare the linkage between IR and CMM across obesity phenotypes, we used the PAF and SHAP values. Within a causal “what-if” framework, PAF estimates the number or proportion of cases that might be avoided if IR were reduced to an ideal level, reflecting the potential disease burden linked to this exposure (25). SHAP values from the machine-learning model express each prediction as the sum of additive contributions from individual variables; when summarized across participants, they rank variables by importance while retaining the direction of effects and highlighting marginal contributions from interactions (40). This approach facilitated the identification of clinical features whose contributions differed across obesity phenotypes.

Adipose tissue serves a dual role, functioning both as an energy reservoir and as an active endocrine organ. It secretes various bioactive adipokines, which are pivotal regulators of systemic metabolism and inflammation (41, 42). Under physiological conditions, adipokines such as adiponectin enhance insulin sensitivity and suppress inflammation (43). However, in the context of obesity-associated IR, adipose tissue undergoes substantial remodeling characterized by adipocyte hypertrophy, hypoxia, fibrosis, and the infiltration of pro-inflammatory immune cells, notably M_1_ macrophages (44, 45). This state, referred to as adipose tissue dysfunction, precipitates a profound shift in adipokine secretion: adiponectin levels, which confer anti-inflammatory and insulin-sensitizing effects, are significantly reduced, whereas the secretion of pro-inflammatory and IR-promoting factors, including leptin (often accompanied by leptin resistance), resistin, retinol-binding protein 4, and tumor necrosis factor-alpha, is markedly elevated (46). This adipokine imbalance might critically contribute to the pathogenesis and progression of CMM. Diminished adiponectin attenuates its protective effects on endothelial function and its role in improving insulin sensitivity in the liver and skeletal muscle (44, 47, 48). Concurrently, elevated pro-inflammatory adipokines exacerbate systemic chronic low-grade inflammation and act directly on vasculature, liver, and cardiac tissue, promoting endothelial dysfunction, hepatic lipogenesis, and cardiac remodeling (49). Consequently, dysfunctional adipose tissue serves as a persistent source of adverse systemic signaling that drives the individual components of CMM.

More importantly, compared to general adiposity (captured by BMI), adipose tissue around the abdomen, particularly visceral adiposity (reflected by WC), is accompanied by more pronounced pathophysiological mechanisms, including chronic systemic inflammation, IR, and endothelial dysfunction, all of which directly promote atherosclerosis and metabolic dysregulation (50). Clinically, abdominal obesity independently predicts higher risks of coronary artery disease, heart failure, atrial fibrillation, and sudden cardiac death, even in individuals with normal BMI (50). Therefore, given its potency as a modifiable risk factor, waist-centered assessment should be a cornerstone of strategies aimed at both cardiovascular risk stratification and the development of targeted interventions (50).

Given that abdominal obesity, rather than general obesity, is a pronounced driver of cardiovascular diseases (50), and considering that IR is widely recognized as the core pathophysiological mechanism underlying the component diseases of CMM (10–12), it is biologically plausible that the magnitude of IR’s contribution to CMM risk varies across different newly defined obesity subgroups, with abdominal obesity weighing more heavily than the classic category. Our study provides clinical epidemiological evidence supporting this hypothesis, demonstrating that the linkage between surrogate IR markers (TyG index and eGDR) and CMM incidence is most pronounced in the predominantly isolated central obesity group. This suggests that the pathophysiological impact of adipose tissue dysfunction and resultant IR may be particularly salient in this distinct subpopulation.

Beyond the primary analysis across redefined obesity categories, we performed stratified analyses by age and sex within each obesity subgroup. Notably, in the no obesity-risk and dual obesity-risk subgroups, when stratified by age, the inverse association between eGDR and CMM risk was significantly stronger in participants aged <65 years, whereas it was notably attenuated in those older than 65 years (*p* for interaction < 0.05). This suggests that the predictive capacity of IR for CMM demonstrates significant age-dependent variability. This implies that strategies aimed at preventing CMM through the modulation of insulin sensitivity may need to consider not only an individual’s specific obesity subgroup but also these demographic factors. The attenuated effect observed in older adults could reflect competing risks, the influence of longer-term comorbidities, or age-related alterations in metabolism and body composition. While these subgroup observations generate important hypotheses, they require validation in independent, prospectively designed cohorts to confirm their reproducibility and elucidate the underlying biological or clinical drivers.

## Strengths

Our study has several strengths. First, we attempt to apply a newly refined obesity classification to demonstrate the heterogeneous linkage between surrogate markers of IR (TyG index and eGDR) and incident CMM across different obesity subgroups. Second, by quantifying the PAF within each obesity subgroup, we provide an epidemiological estimate of the potential reduction in CMM burden achievable by improving insulin sensitivity, thereby offering a data-driven foundation for identifying subpopulations that would benefit most from targeted preventive strategies. Third, the analysis leverages a large, well-characterized prospective cohort, enhancing the statistical power and generalizability of our findings. Finally, we built separate machine-learning prediction models for each obesity subgroup and calculated SHAP values to enable a clear, side-by-side comparison of each feature’s importance within the models.

## Limitations

Our study has several limitations. First, the cohort was exclusively composed of Europeans. Generalizability may be limited to individuals of Asian, African, or other ethnic backgrounds, as differences in genetic predisposition, body composition, and metabolic profiles may influence the observed relationships. Future studies involving diverse, multi-ethnic cohorts are essential to validate and extend our conclusions. Second, while our models accounted for an extensive set of potential confounding variables, the possibility of residual or unmeasured confounding cannot be entirely ruled out, which might still bias our results. Third, a substantial proportion of participants were excluded because of missing data required to calculate the insulin resistance indices, resulting in a potentially healthier and more selected analytic sample. This may have underestimated the absolute incidence of CMM, limited generalizability, and attenuated the observed associations, although the direction of selection bias cannot be determined with certainty. Fourth, despite utilizing the prospective cohort data, our study design remains observational in nature. Consequently, while we established a temporal sequence between IR-related markers and CMM onset, the associations we observed should not be interpreted as definitive proof of causality. Experimental or interventional studies are required to confirm a direct causal link between improving insulin sensitivity and reducing CMM risk in the newly redefined obesity subgroups. Fifth, although death was a competing event for CMM and could affect risk estimates, deaths were rare in this cohort, and some obesity-phenotype subgroups had no deaths. These findings therefore need confirmation in larger populations. Sixth, although covariates were imputed using a random forest–based method, its relatively high missingness may still have introduced residual bias. Seventh, because hypertension status and HbA1c are incorporated into eGDR, and fasting glucose is included in the TyG index, partial overlap exists between these insulin resistance surrogates and the CMM definition, which includes hypertension and diabetes. This overlap may have strengthened the observed associations, particularly for eGDR, and the corresponding findings should therefore be interpreted cautiously. Eighth, cardiometabolic conditions were predominantly self-reported, and insulin resistance surrogates were based on single measurements. This may have led to systematic misclassification, including missed undiagnosed hypertension or diabetes, and limited the assessment of long-term metabolic status.

## Conclusion

In this study, the TyG index and eGDR were strong predictors of new-onset CMM. The association varied across our refined obesity categories and was strongest in the predominantly isolated central obesity group. Age-stratified analyses further suggest that risk patterns differ across age groups. Incorporating IR markers into clinical assessment, particularly among individuals with the predominantly isolated central obesity phenotype, may improve the identification of high-risk profiles and inform future research on phenotype-tailored strategies to improve metabolic health and reduce CMM risk.

## Data Availability

The datasets analyzed during this study are accessible on the ELSA website (https://www.elsa-project.ac.uk).

## Abbreviations

AUC: area under the curve
BMI: body mass index
CMM: cardiometabolic multimorbidity
CI: confidence interval
DM: diabetes mellitus
eGDR: estimated glucose disposal rate
ELSA: English longitudinal study of ageing
FPG: fasting blood glucose
HbA1c: glycosylated haemoglobin
HDL-C: high-density lipoprotein cholesterol
HLD: hyperlipidemia
HR: hazard ratio
IQR: interquartile range
IR: insulin resistance
LDL-C: low-density lipoprotein cholesterol
LE8: life’s essential 8
NPV: negative predictive value
K-M: Kaplan–Meier
PAF: population attributable fraction
PPV: positive predictive value
RCS: restricted cubic spline
ROC: receiver operating characteristic
SD: standard deviation
SHAP: Shapley additive explanations
TC: total cholesterol
TG: triglyceride
TyG: triglyceride–glucose
T2DM: type 2 diabetes
VIF: variance inflation factor
WC: waist circumference
XGBoost: extreme gradient boosting
