# Supplementary figures and images for: The associations of the triglyceride–glucose index and estimated glucose disposal rate with incident cardiometabolic multimorbidity vary across obesity phenotypes: a longitudinal cohort study
Source: Front Nutr. 2026 Jul 9;13:1805727. doi: 10.3389/fnut.2026.1805727 (PMC13391553; doi:10.3389/fnut.2026.1805727)

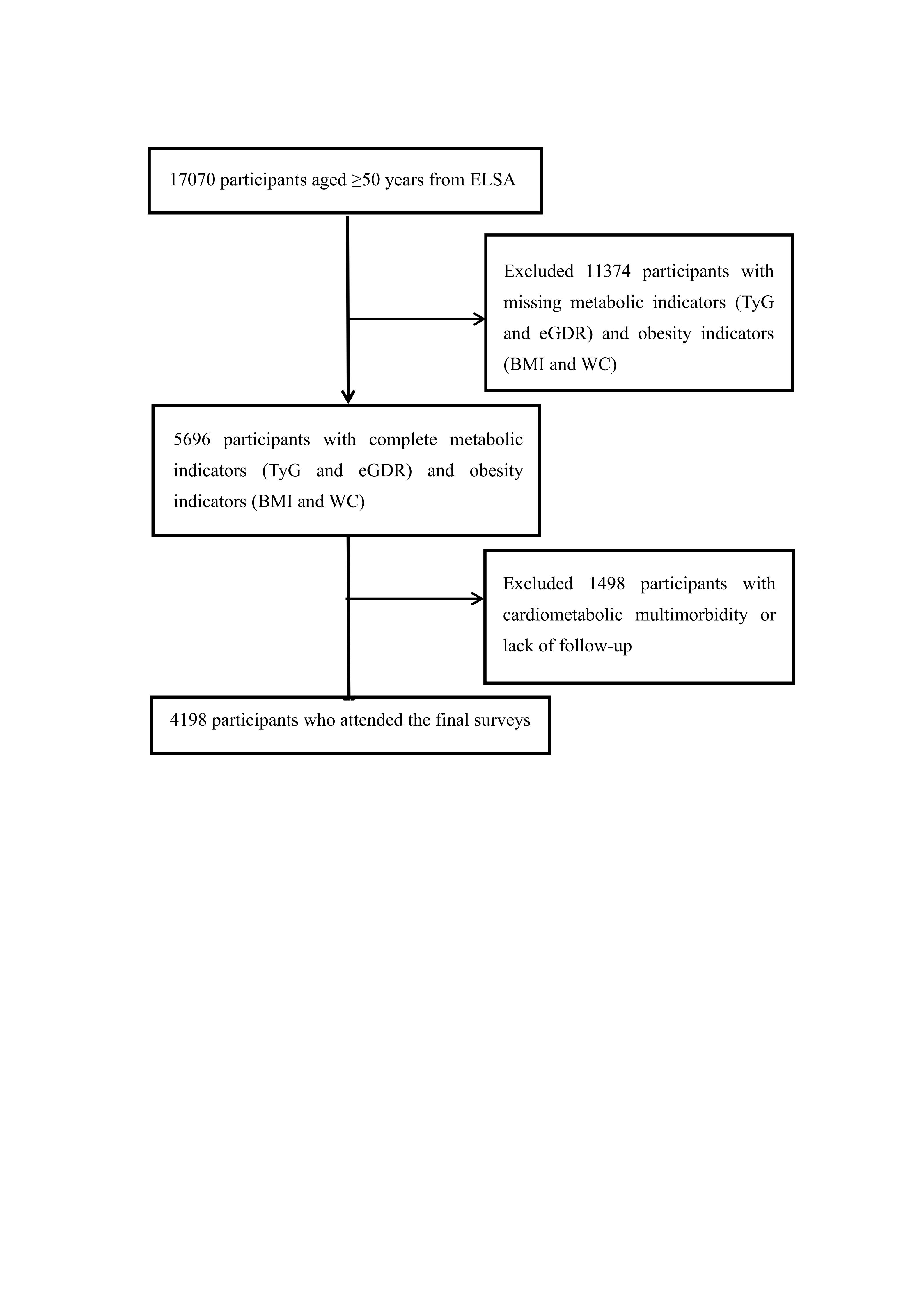

Supplement: Supplementary file 1 [file Image_1.JPEG]

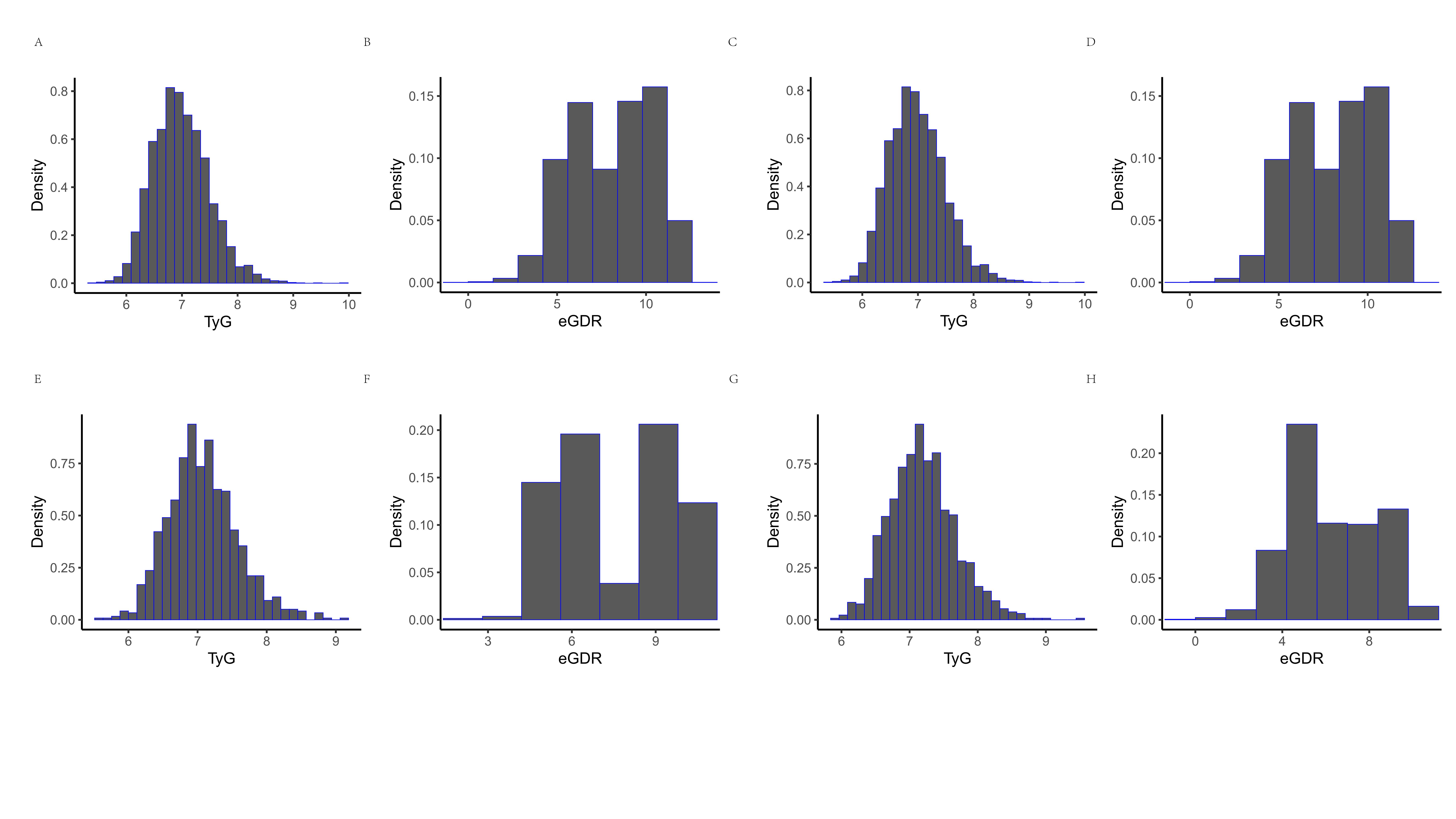

Supplement: Supplementary file 2 [file Image_2.JPEG]

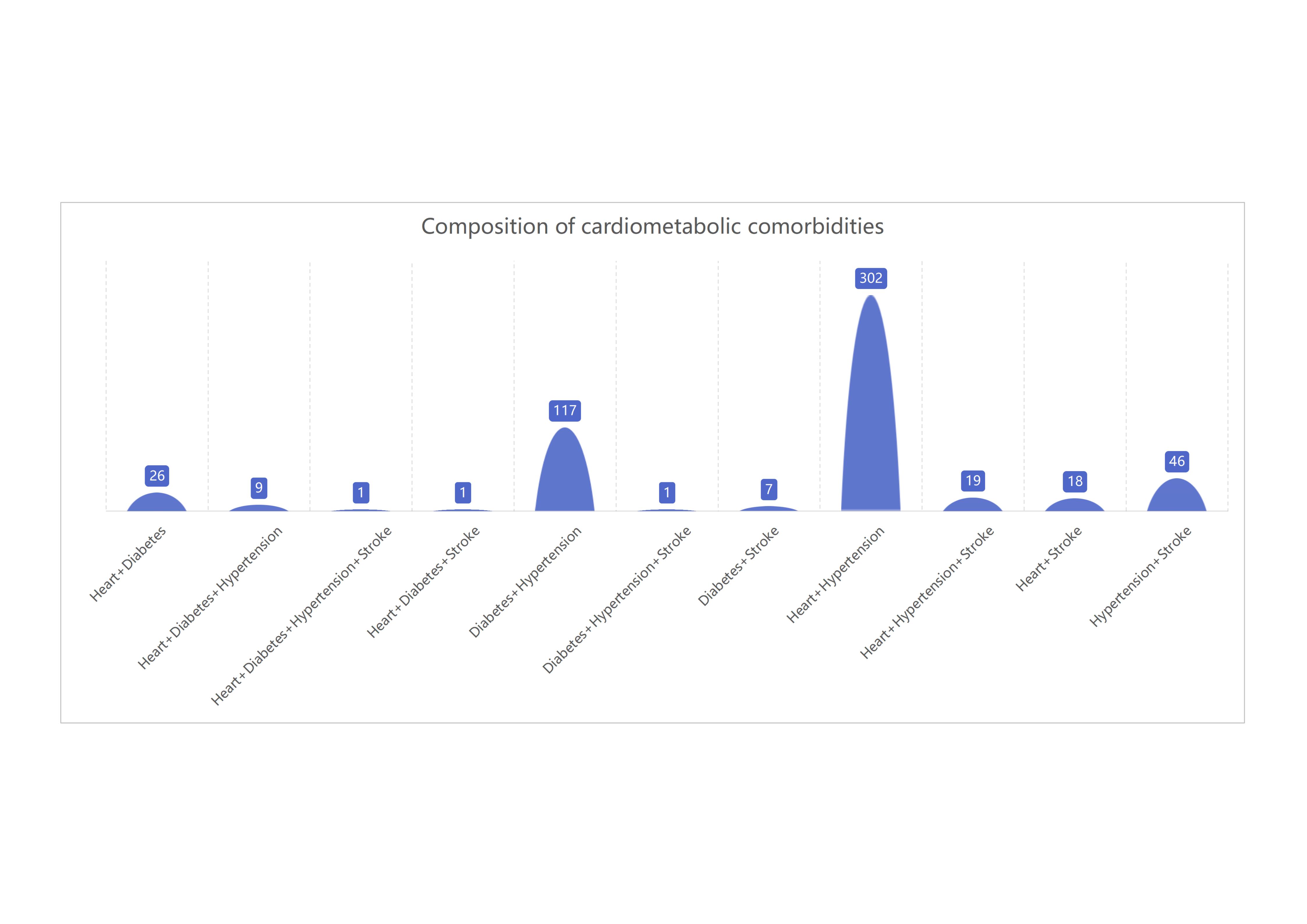

Supplement: Supplementary file 3 [file Image_3.JPEG]

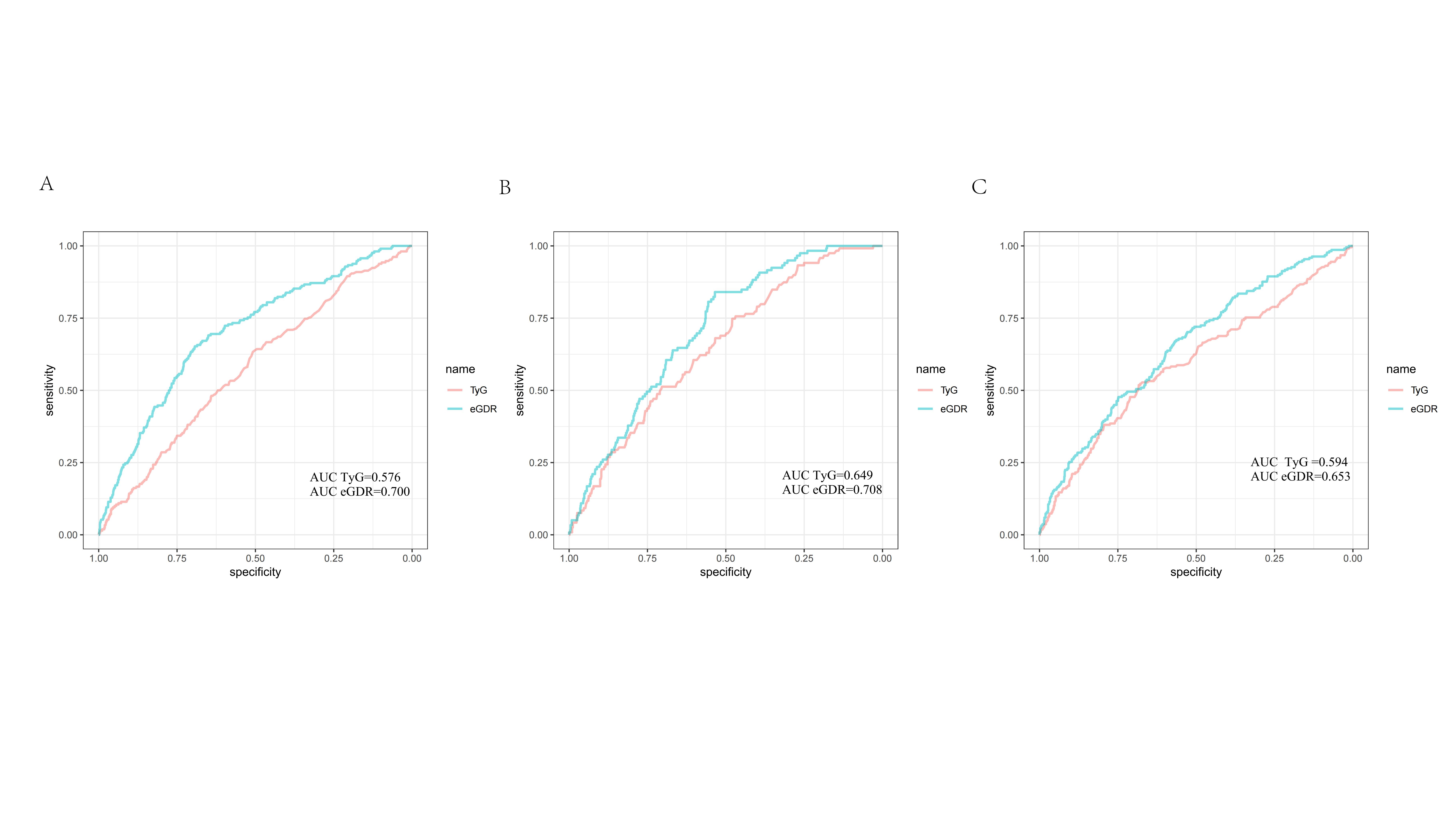

Supplement: Supplementary file 4 [file Image_4.JPEG]

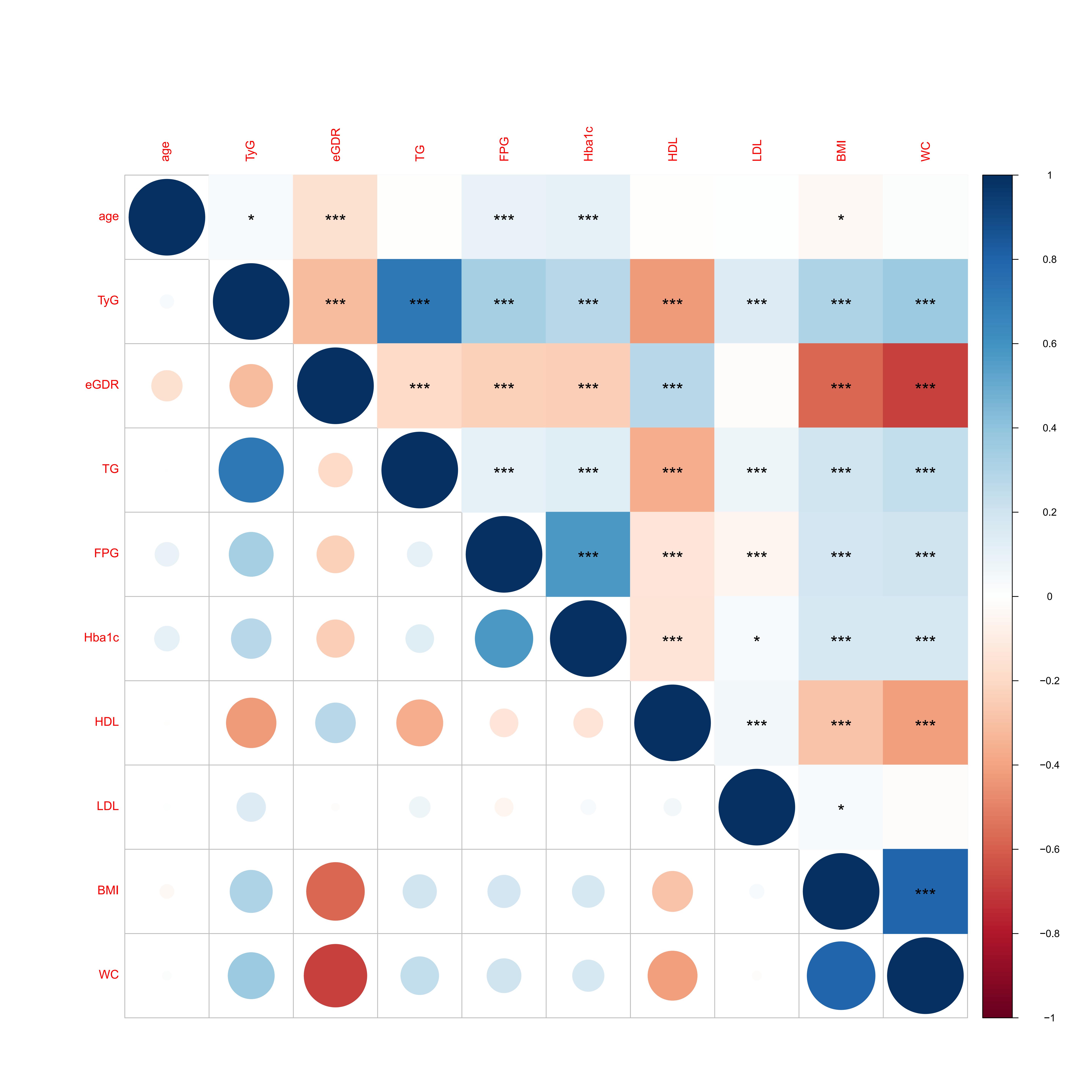

Supplement: Supplementary file 5 [file Image_5.JPEG]
