# Supplementary material for: The associations of the triglyceride–glucose index and estimated glucose disposal rate with incident cardiometabolic multimorbidity vary across obesity phenotypes: a longitudinal cohort study
Source: Front Nutr. 2026 Jul 9;13:1805727. doi: 10.3389/fnut.2026.1805727 (PMC13391553; doi:10.3389/fnut.2026.1805727)
Supplement: Supplementary file 6 [file Table_1.DOCX]

**Supplementary Materials**

**The association between insulin resistance indices (TyG index and eGDR) and incident cardiometabolic multimorbidity varies across obesity phenotypes: A longitudinal cohort study**

Chenyang Li, Yu Feng, Ying Tang, Yupu Shao, Xiaoqin Luo, Yifan Chen, Jiafeng Lin, Shengyuan Gu

**Supplementary Methods**

**Supplementary Table 1** Obesity risk group definitions based on BMI and WC.

**Supplementary Table 2** Baseline characteristics of included and excluded participants.

**Supplementary Table 3** The number and proportion of missing values for each covariate.

**Supplementary Table 4** Multivariable associations between metabolic indicators (TyG and eGDR Index) and risk of CMM.

**Supplementary Table 5** VIFs for covariates in Cox regression models of CMM.

**Supplementary Figure 1** Flow diagram for participants included in the study.

**Supplementary Figure 2** Distribution of key insulin resistance indicators (TyG and eGDR). **A-B:** No obesity-risk group. **C-D:** Predominantly isolated central obesity. **E-F:** Dual obesity-risk group.

**Supplementary Figure 3** Composition of CMM.

**Supplementary Figure 4** ROC curves for predicting CMM. **A:** No obesity-risk group. **B:** Predominantly isolated central obesity. **C:** Dual obesity-risk group.

**Supplementary Figure 5** Pearson Correlation Heatmap of Baseline Variables.

**Supplementary Methods**

**Metabolic index calculation formula**

In even-numbered waves, ELSA conducts venous blood sampling during home visits by trained nurses, and all specimens are then analyzed at the Royal Victoria Infirmary (Newcastle upon Tyne, UK). This protocol ensures standardized biochemical assessment and supports high data quality across waves.

$$TyG=\ln\left[ {TG(mg/dL)\times FPG(mg/dL)}/2 \right]$$

$$eGDR=21.158-(0.09\times WC(cm))-(3.407\times HTN(yes=1/no=0))-(0.551\times HbA1c(percentage))$$

**CMM**

In this analysis, cardiometabolic multimorbidity (CMM) was defined as the coexistence of at least two cardiometabolic conditions, including hypertension, diabetes, heart disease, or stroke. In the English Longitudinal Study of Ageing (ELSA), these conditions were identified through standardized self-reported physician diagnoses collected during nurse visits. Participants were asked, “Has a doctor ever told you that you have high blood pressure or hypertension?”; “Has a doctor ever told you that you have diabetes or high blood sugar?”; and “Has a doctor ever told you that you have heart disease such as myocardial infarction, coronary heart disease, angina, heart failure, or another heart problem?” or “Have you ever been told by a doctor that you have had a stroke?” Information on medication use for these conditions was also considered. Diabetes status was further validated by biochemical measurements (fasting plasma glucose ≥126 mg/dL or HbA1c ≥ 6.5%) obtained in the nurse visit subsample. Participants were classified as having developed CMM when a second cardiometabolic condition was first identified. The time to CMM onset was calculated as the interval between the preceding wave and the first wave in which CMM was recorded.

**Statistical analysis**

Continuous variables were summarized according to their distribution, using mean ± standard deviation (SD) for approximately normally distributed data and median (interquartile range (IQR)) otherwise. Group comparisons used parametric tests (two-sample t-test or one-way ANOVA) for normally distributed data and rank-based tests for non-normally distributed data (Wilcoxon rank-sum test for two groups; Kruskal–Wallis test for more than two groups). Categorical variables are presented as counts (percentages) and compared using the χ² test. Missing covariate data were handled under a missing-at-random assumption using multiple imputation by chained equations.

**Supplementary Table 1.** Obesity risk group definitions based on BMI and WC.

| **Obesity risk group** | **BMI criterion (kg/m²)** | **WC criterion (cm; sex-specific)** | **Classification rule** |
| --- | --- | --- | --- |
| No obesity risk | BMI < 30kg/m² | Men < 102cm; Women < 88cm | Both BMI and WC are below the thresholds |
| Predominantly isolated central obesity | BMI ≥30 kg/m² | Men < 102cm; Women < 88cm | Exactly one criterion is met (BMI elevated OR WC elevated) |
|  | BMI < 30kg/m² | Men ≥ 102cm; Women ≥ 88cm |  |
| Dual obesity-risk | BMI > 30kg/m² | Men ≥ 102cm; Women ≥ 88cm | Both BMI and WC meet/exceed the thresholds |

Abbreviations: BMI, body mass index; WC, waist circumference.

**Supplementary Table 2.** Baseline characteristics of included and excluded participants.

| **Variables** | **Total (n = 17068)** | **Complete (n = 4198)** | **Missing (n = 12870)** | ***P* value** |
| --- | --- | --- | --- | --- |
| age, Median (IQR) | 65.0 (58.0, 75.0) | 63.0 (58.0, 70.0) | 66.0 (58.0, 77.0) | **< 0.001** |
| **gender, n (%)** |  |  |  | **0.001** |
| Male | 7926 (46.4) | 1860 (44.3) | 6066 (47.1) |  |
| Female | 9142 (53.6) | 2338 (55.7) | 6804 (52.9) |  |
| **Education, n (%)** |  |  |  | **< 0.001** |
| Less than high school | 7046 (45.2) | 1250 (32.3) | 5796 (49.4) |  |
| High school or equivalent | 2952 (18.9) | 900 (23.3) | 2052 (17.5) |  |
| College or above | 5606 (35.9) | 1720 (44.4) | 3886 (33.1) |  |
| **Marital, n (%)** |  |  |  | **< 0.001** |
| Married or partnered | 7631 (71.0) | 3136 (74.7) | 4495 (68.6) |  |
| Divorced or never partnered | 3120 (29.0) | 1062 (25.3) | 2058 (31.4) |  |
| **Smoke, n (%)** |  |  |  | **< 0.001** |
| No | 9066 (85.9) | 3667 (87.8) | 5399 (84.7) |  |
| Yes | 1487 (14.1) | 510 (12.2) | 977 (15.3) |  |
| **Drink, n (%)** |  |  |  | **< 0.001** |
| No | 2892 (33.0) | 1044 (27.4) | 1848 (37.4) |  |
| ≥ 1 days/week | 5859 (67.0) | 2771 (72.6) | 3088 (62.6) |  |
| **Moderate physical activity, n (%)** |  |  |  | **< 0.001** |
| ≥ 1 days/week | 6405 (59.6) | 2930 (69.8) | 3475 (53.1) |  |
| < 1 days/week | 4338 (40.4) | 1268 (30.2) | 3070 (46.9) |  |
| **Vigorous physical activity, n (%)** |  |  |  | **< 0.001** |
| ≥ 1 days/week | 2082 (19.4) | 1025 (24.4) | 1057 (16.1) |  |
| < 1 days/week | 8661 (80.6) | 3173 (75.6) | 5488 (83.9) |  |
| BMI, Median (IQR) | 27.3 (24.7, 30.7) | 26.9 (24.5, 30.2) | 27.6 (24.8, 31.2) | **< 0.001** |
| WC, Median (IQR) | 95.7 (86.6, 104.7) | 94.0 (85.5, 102.7) | 96.8 (87.3, 106.2) | **< 0.001** |
| TyG, Median (IQR) | 7.0 (6.7, 7.3) | 7.0 (6.6, 7.3) | 7.1 (6.7, 7.5) | **< 0.001** |
| eGDR, Median (IQR) | 7.3 (5.7, 9.6) | 8.4 (6.1, 10.0) | 6.7 (5.3, 9.0) | **< 0.001** |
| TG, Median (IQR) | 1.5 (1.1, 2.1) | 1.4 (1.0, 1.9) | 1.6 (1.2, 2.3) | **< 0.001** |
| FPG, Median (IQR) | 4.8 (4.5, 5.1) | 4.8 (4.5, 5.1) | 4.9 (4.5, 5.4) | **< 0.001** |
| Hba1c, Median (IQR) | 5.8 (5.5, 6.0) | 5.7 (5.5, 5.9) | 5.8 (5.6, 6.2) | **< 0.001** |
| HDL, Median (IQR) | 1.5 (1.3, 1.8) | 1.5 (1.3, 1.8) | 1.4 (1.2, 1.7) | **< 0.001** |
| LDL, Median (IQR) | 3.6 (2.9, 4.2) | 3.8 (3.2, 4.4) | 3.4 (2.7, 4.1) | **< 0.001** |
| **Depression, n (%)** |  |  |  | **< 0.001** |
| No | 8746 (85.2) | 3746 (89.7) | 5000 (82) |  |
| Yes | 1525 (14.8) | 428 (10.3) | 1097 (18) |  |
| **Cancer, n (%)** |  |  |  | **0.013** |
| No | 9892 (92.0) | 3897 (92.9) | 5995 (91.5) |  |
| Yes | 855 ( 8.0) | 300 (7.1) | 555 (8.5) |  |
| **Lung, n (%)** |  |  |  | **< 0.001** |
| No | 10138 (94.3) | 4015 (95.7) | 6123 (93.5) |  |
| Yes | 609 ( 5.7) | 182 (4.3) | 427 (6.5) |  |
| **Arthritis, n (%)** |  |  |  | **< 0.001** |
| No | 7031 (65.4) | 2838 (67.6) | 4193 (64) |  |
| Yes | 3715 (34.6) | 1359 (32.4) | 2356 (36) |  |

A *P* value < 0.05 indicated a significant difference.

Abbreviations: BMI, body mass index; eGDR, estimated glucose disposal rate; FPG, fasting blood glucose; Hba1c, glycosylated hemoglobin; HDL, high-density lipoprotein; LDL, low-density lipoprotein; IQR, interquartile range; TG, triglyceride; TyG, triglyceride-glucose; WC, waist circumference.

**Supplementary Table 3.** The number and proportion of missing values for each covariate.

| **Characteristic** | **Non- Missing** | **Missing** | **Missing proportion** |
| --- | --- | --- | --- |
| marital status | 4198 | 0 | 0 |
| education | 3870 | 328 | 7.8% |
| smoke | 4177 | 21 | 0.5% |
| drink | 3815 | 383 | 9.1% |
| vigorous physical activity | 4198 | 0 | 0 |
| moderate physical activity | 4198 | 0 | 0 |
| depression | 4174 | 24 | 0.5% |
| cancer | 4197 | 1 | 0.1% |
| lung | 4197 | 1 | 0.1% |
| arthritis | 4197 | 1 | 0.1% |
| Medication for HBP | 4197 | 1 | 0.1% |
| Medication for DM | 4198 | 0 | 0 |
| Medication for HLD | 4194 | 4 | 0.1% |
| HDL | 3787 | 411 | 9.7% |
| LDL | 2600 | 1598 | 38% |

Abbreviations: DM, diabetes mellitus; HBP, hypertension; HDL, high-density lipoprotein; HLD, hyperlipidemia; LDL, low-density lipoprotein.

**Supplementary Table 4.** Multivariable associations between metabolic indicators (TyG and eGDR Index) and risk of CMM.

| **Indices** | **Groups** | **Number** | **Model 1** | **Model 2** | **Model 3** |
| --- | --- | --- | --- | --- | --- |
|  |  |  | **HR (95% CI) *P* value** | **HR (95% CI) *P* value** | **HR (95% CI) *P* value** |
| **No obesity-risk group** | | | | | |
| TyG | Continuous | N = 1580 | **1.251 (1.057–1.481) 0.009** | **1.207 (1.007–1.448) 0.041** | **1.218 (1.014–1.463) 0.034** |
|  | Q1 | N = 395 | 1(Ref) | 1(Ref) | 1(Ref) |
|  | Q2 | N = 399 | 1.01 (0.597–1.730) 0.956 | 0.85 (0.482–1.500) 0.577 | 0.89 (0.505–1.580) 0.699 |
|  | Q3 | N = 393 | 1.09 (0.650–1.840) 0.739 | 0.95 (0.554–1.660) 0.881 | 0.99 (0.574–1.730) 0.986 |
|  | Q4 | N = 393 | 1.54 (0.948–2.510) 0.080 | 1.33 (0.793–2.220) 0.281 | 1.38 (0.822–2.320) 0.222 |
|  | *P* for trend |  | **0.061** | **0.185** | **0.155** |
| eGDR | Continuous | N = 1580 | **0.484 (0.396–0.590) < 0.001** | **0.492 (0.399–0.608) < 0.001** | **0.495 (0.401–0.612) < 0.001** |
|  | Q1 | N = 395 | 1(Ref) | 1(Ref) | 1(Ref) |
|  | Q2 | N = 395 | **0.39 (0.255–0.616) < 0.001** | **0.36 (0.222–0.583) < 0.001** | **0.52 (0.372–0.730) < 0.001** |
|  | Q3 | N = 395 | **0.27 (0.166–0.466) < 0.001** | **0.30 (0.178–0.520) < 0.001** | **0.32 (0.208–0.487) < 0.001** |
|  | Q4 | N = 395 | **0.17 (0.086–0.344) < 0.001** | **0.19 (0.095–0.392) < 0.001** | **0.28 (0.165–0.472) < 0.001** |
|  | *P* for trend |  | **< 0.001** | **< 0.001** | **< 0.001** |
| **Predominantly isolated central obesity** | | | | | |
| TyG | Continuous | N = 773 | **1.583 (1.267–1.978) < 0.001** | **1.456 (1.145–1.854) 0.002** | **1.496 (1.174–1.906) < 0.001** |
|  | Q1 | N = 196 | 1(Ref) | 1(Ref) | 1(Ref) |
|  | Q2 | N = 193 | 1.88 (0.875–4.060) 0.106 | 1.71 (0.787–3.710) 0.176 | 1.64 (0.751–3.580) 0.215 |
|  | Q3 | N = 191 | 1.86 (0.855–4.040) 0.118 | 1.63 (0.740–3.600) 0.225 | 1.74 (0.787–3.860) 0.171 |
|  | Q4 | N = 193 | **3.54 (1.730–7.240) < 0.001** | **2.73 (1.300–5.710) 0.007** | **2.83 (1.350–5.940) 0.005** |
|  | *P* for trend |  | **< 0.001** | **0.008** | **0.004** |
| eGDR | Continuous | N = 773 | **0.369 (0.265–0.514) < 0.001** | **0.378 (0.270–0.529) < 0.001** | **0.369 (0.262–0.519) < 0.001** |
|  | Q1 | N = 194 | 1(Ref) | 1(Ref) | 1(Ref) |
|  | Q2 | N = 193 | 1.26 (0.574–2.780) 0.563 | 1.44 (0.646–3.230) 0.370 | 1.37 (0.614–3.050) 0.443 |
|  | Q3 | N = 193 | **0.24 (0.113–0.540) < 0.001** | **0.23 (0.103–0.546) 0.007** | **0.23 (0.099–0.534) < 0.001** |
|  | Q4 | N = 193 | **0.15 (0.045–0.538) 0.003** | **0.13 (0.033–0.528) < 0.001** | **0.12 (0.032–0.502) 0.003** |
|  | *P* for trend |  | **< 0.001** | **< 0.001** | **< 0.001** |
| **Dual obesity-risk group** | | | | | |
| TyG | Continuous | N = 839 | **1.319 (1.130–1.541) < 0.001** | **1.189 (1.000–1.414) 0.049** | 1.173 (0.984–1.398) 0.073 |
|  | Q1 | N = 216 | 1(Ref) | 1(Ref) | 1(Ref) |
|  | Q2 | N = 204 | 0.83 (0.504–1.360) 0.463 | 0.82 (0.491–1.380) 0.454 | 0.72 (0.429–1.210) 0.216 |
|  | Q3 | N = 209 | 1.14 (0.712–1.820) 0.588 | 0.99 (0.605–1.620) 0.968 | 0.96 (0.587–1.570) 0.872 |
|  | Q4 | N = 210 | **1.71 (1.110–2.640) 0.015** | 1.30 (0.815–2.070) 0.271 | 1.17 (0.730–1.870) 0.519 |
|  | *P* for trend |  | **0.005** | **0.181** | **0.288** |
| eGDR | Continuous | N = 839 | **0.508 (0.418–0.617) < 0.001** | **0.483 (0.375–0.622) < 0.001** | **0.472 (0.365–0.611) < 0.001** |
|  | Q1 | N = 210 | 1(Ref) | 1(Ref) | 1(Ref) |
|  | Q2 | N = 210 | **0.65 (0.444–0.959) 0.029** | **0.60 (0.400–0.913) 0.016** | **0.57 (0.383–0.876) 0.009** |
|  | Q3 | N = 210 | **0.42 (0.271–0.655) < 0.001** | **0.41 (0.252–0.667) < 0.001** | **0.40 (0.251–0.667) < 0.001** |
|  | Q4 | N = 209 | **0.21 (0.120–0.384) < 0.001** | **0.22 (0.124–0.415) < 0.001** | **0.21 (0.116–0.391) < 0.001** |
|  | *P* for trend |  | **< 0.001** | **< 0.001** | **< 0.001** |

A *P* value < 0.05 indicated a significant difference.

Abbreviations: CMM, cardiometabolic multimorbidity; DM, diabetes mellitus; eGDR, estimated glucose disposal rate; HBP, hypertension; HDL, high-density lipoprotein; HLD, hyperlipidemia; LDL, low-density lipoprotein; TyG, triglyceride-glucose.

Model 1: Adjusted for age, sex, smoking status, and alcohol consumption.

Model 2: Adjusted for age, sex, smoking status, alcohol consumption, marital status, education, vigorous physical activity, and moderate physical activity.

Model 3: Adjusted for age, sex, marital status, education, smoking status, alcohol consumption, vigorous physical activity, moderate physical activity, LDL, HDL, medication for HBP, medication for DM, medication for HLD, lung disease, cancer, arthritis and depression.

**Supplementary Table 5.** VIFs for covariates in Cox regression models of CMM.

| **Variables** | **VIF for CMM** |
| --- | --- |
| Sex | 2.233 |
| Age | 1.195 |
| Marital status | 1.123 |
| Education level | 1.131 |
| Smoking status | 1.096 |
| Alcohol consumption | 1.159 |
| Vigorous activity | 1.095 |
| Moderate activity | 1.114 |
| BMI | 5.039 |
| WC | 5.467 |
| Cancer | 1.019 |
| Lung disease | 1.063 |
| Depression | 1.086 |
| Arthritis | 1.094 |
| Medication for HBP | 1.161 |
| Medication for DM | 1.036 |
| Medication for HLD | 1.132 |
| LDL | 1.034 |
| HDL | 1.342 |

Bold values indicate multicollinearity between variables.

Abbreviations: BMI, body mass index; CMM, cardiometabolic multimorbidity; DM, diabetes mellitus; HBP, hypertension; HDL, high-density lipoprotein; HLD, hyperlipidemia; LDL, low-density lipoprotein; VIF, v**ariance inflation factor**; WC, waist circumference.
